# Treatment adherence and blood pressure outcome among hypertensive out-patients in two tertiary hospitals in Sokoto, Northwestern Nigeria

**DOI:** 10.1186/s12872-018-0934-x

**Published:** 2018-10-19

**Authors:** Rasaq Adisa, Olumide Ayodeji Ilesanmi, Titilayo Oyelola Fakeye

**Affiliations:** 1 Department of Clinical Pharmacy & Pharmacy Administration, Faculty of Pharmacy, University of Ibadan, Ibadan, Nigeria; 2 Pharmacy Department, Usmanu Danfodiyo University Teaching Hospital, Sokoto, Sokoto state Nigeria

**Keywords:** Treatment adherence, Ambulatory hypertensive patients, Blood pressure, Non-pharmacological measures

## Abstract

**Background:**

Treatment adherence play important roles in blood pressure control leading to reduction in morbidity and mortality. This study therefore assessed adherence to pharmacological and non-pharmacological therapies among ambulatory hypertensive patients. Reasons for treatment non-adherence, and association between adherence and blood pressure were also investigated.

**Methods:**

Cross-sectional questionnaire-guided interview and retrospective review of medical records of 605-patients from two-tertiary healthcare institutions in Sokoto, Northwestern Nigeria. Nine-item modified Morisky adherence scale was used to assess medication adherence. Overall adherence score to lifestyle modifications was obtained from the total scores from 4-domains of non-pharmacological measures including cigarette smoking and alcohol cessation, salt-restriction and exercise. Patient-specific adherence education was provided at contact to resolve the knowledge gap(s). Clinical-parameters were retrieved at contact and subsequent 2-months appointment. Descriptive statistics, Chi-square and Student’s t-test were used for analysis at *p* < 0.05.

**Results:**

Fifty-four (8.9%) patients were adherent to medications. Forgetfulness (404; 35.2%) was the most common reason for medication non-adherence. Use of buddy/companion reminder (605, 30.2%) top the list of adherence education. Overall adherence to lifestyle modifications was 36(6.0%). Mean systolic blood pressure (SBP) at contact was 149.6 ± 22.5 versus 134.2 ± 15.8 mmHg at 2-months with a 10% reduction. There were significant associations in baseline SBP for patients with or without adherence to medication, cigarette smoking cessation, and exercise (*p* < 0.05).

**Conclusions:**

Overall adherence to antihypertensive medications and lifestyle modifications is suboptimal, underscoring the need for continuous patient-specific adherence education to ensure better therapeutic outcomes.

**Electronic supplementary material:**

The online version of this article (10.1186/s12872-018-0934-x) contains supplementary material, which is available to authorized users.

## Background

Hypertension is a common cardiovascular disease worldwide, contributing 4.5% of the global disease burden and 12.8% premature deaths annually [1, 2]. According to the World Health Organization, more than 80% of deaths from hypertension and associated cardiovascular diseases occur in low and middle-income countries, particularly among people of low socioeconomic status [3–5]. In 2008, the WHO estimated hypertension prevalence of 42.8% in Nigeria [6, 7], the high prevalence is believed to be largely due to an increasing adult population and rapid urbanisation, overweight and obesity, physical inactivity [7, 8], as well as uptake of western lifestyle including high consumption of processed foods and increased tobacco and alcohol intake [9].

Despite recent advances in drug therapy, majority of diagnosed hypertensive patients are poorly controlled [4, 10, 11]. Reasons for inadequate control of hypertension are heterogeneous including low adherence to antihypertensive medications and lifestyle changes, low compliance with scheduled follow-up visits and suboptimal pharmacotherapy [12, 13]. Studies have shown that compliance with lifestyle modifications such as regular exercise for at least 30 min thrice per week, eating salt and fat free diets, cessation of smoking, and reduction in daily alcohol consumption are essential for adequate lowering of blood pressure [14, 15]. However, in many developed and developing countries, non-adherence with antihypertensive medications and lifestyle recommendations remain a serious problem [7, 16, 17]. It is estimated that more than 70% of patients on antihypertensive medications do not take them as prescribed [18–20]. The non-adherence practice may be particularly higher in developing countries where there is poor accessibility to medicines and healthcare services [13, 21–23], as well as low level of awareness about the lifelong nature of hypertension management among patients [24]. Optimal control of blood pressure has been reported to reduce the incidence of stroke by an average of 35–40%, myocardial infarction by 20–25% and heart failure by > 50% [25, 26]. Thus, adoption of healthy lifestyle as well as ensuring regular and continuous adherence to prescribed medications are integral to successful management of hypertension [16, 22, 27]. In Nigeria and other resource-poor countries, studies have focused more on assessment of compliance to pharmacotherapy [28–30], while evidence-based research evaluating adherence to non-pharmacological measures is scarce.

### Aim of the study

This study aimed to comprehensively evaluate adherence to pharmacotherapy and non-pharmacological measures among ambulatory hypertensive patients attending two healthcare institutions in Sokoto, Northwestern Nigeria. Reasons for treatment non-adherence were evaluated, while perception and beliefs about hypertensive management were also explored, with pharmacist-led patient-specific adherence education provided as appropriate to resolve the knowledge gap(s). Association between treatment adherence and blood pressure at contact and the subsequent 2-months clinic appointment were investigated.

## Methods

### Study design

This was a cross-sectional questionnaire-guided interview carried out on 605 consented patients attending the cardiology outpatient clinic of two hospitals between February and May, 2017. A retrospective review of patients’ medical records for disease-specific clinical parameters including blood pressure and prescribed regimen was subsequently done.

### Study sites

Medical outpatient clinic of Usmanu Danfodiyo University Teaching Hospital and the Specialist Hospital, both within Sokoto metropolis, Sokoto state, Northwestern Nigeria. The study sites were notable hospitals for specialised care and treatment of hypertensive patients’ referred from other hospitals and healthcare centres within and outside the region.

### Inclusion/exclusion criteria

Patients aged 18 years and above, with a primary diagnosis of hypertension, and who were on antihypertensive medications for at least 3-months were enrolled. Newly diagnosed patients, in-patients and those who declined participation were excluded from the study.

### Sample size determination

Representative sample size for the study was determined using an estimated prevalence of blood pressure control rate of 24.2% [29] at 5% margin of error and 95% confidence level. Based on these assumptions, sample size for each hospital was calculated to be 281 using sample size formula [31]. Adjusting for a 10% non-response rate, the sample size was 312 for each hospital, giving a total of 624 as target sample size.

### Patients’ sampling and recruitment procedure

Eligible patients attending cardiology clinic of the hospitals on Tuesdays and Thursdays were enrolled on every clinic days using consecutive sampling. Patients were approached for participation while waiting to see the physician on the clinic days. The procedure and objectives of the study were explained to patient individually, after which the written informed consent was obtained from individual patient either by appending signature or thumbprint to signify their intention to participate in the study. The questionnaire was translated from English to Hausa language for those who did not understand English with the assistance of an interpreter, while back-translation to English was subsequently done to ensure response consistency. Only consented patients were administered the questionnaire which took between 25 and 30 min to complete. Anonymity and confidentiality of response were assured, while patients were informed that participation in the study is entirely voluntary.

### Data collection instruments

The main instruments for data collection were pre-tested semi-structured questionnaire and data collection form (Additional file 1). The 45-item questionnaire was divided into six sections. Section A assessed socio-demographic characteristics. Section B explored practice of self-monitoring of blood pressure (SMBP) by patients. Section C contained 9-item modified Morisky Adherence Predictor Scale (MMAPS) [32] to evaluate adherence to antihypertensive medications with a follow-up “Show and Tell” questioning approach [33, 34] to further clarify the actual use of prescribed regimen. A “No” response or “never” to any questions on MMAPS was assigned a score of zero, and a “Yes” response or “once in a while, sometimes, and often” has a score of one. Binary categorisation of scores on MMAPS into adherence (total score < 1) versus non-adherence (total score ≥ 1) was defined in accordance with Adisa et al. (2011) [35]. Section D explored the extent of commitment and adherence to non-pharmacological recommendations. Adherence to non-pharmacological measures was considered as total avoidance of cigarette smoking, alcohol consumption, and dietary salt intake, while engaging in exercise for at least 3-days per week was adjudged adherence, with a score of zero. Overall lifestyle modification adherence score is the sum total of scores from the 4-domains of non-pharmacological measures with binary categorisation of scores < 1 for adherence versus ≥1 as non-adherence [35]. Section E assessed patients’ perception about hypertension, using modified Brief Illness Perception Questionnaire (BIPQ) [36] with a 5-point Likert scale. A score of 0–50% represents the degree to which the illness is perceived as “benign” and 51–100% suggests the extent to which the illness is perceived as “threatening”. Section F which clarified belief about medications comprised 9-item Beliefs about Medicine Questionnaire (BMQ) [37], with five questions in the “specific-necessity” domain, while the remaining four questions referred to the “specific-concern” domain. The BMQ uses 5-point Likert questions ranging from strongly disagree (1) to strongly agree (5). Respondents’ scores on each domain were subsequently converted to percentage, with total scores > 50% indicating “stronger” beliefs about the necessity for taking medicines and “stronger” concern about adverse effects, while scores ≤50% suggesting “weaker” beliefs and concern about necessity and adverse effects.

Patient-specific adherence education was provided for every participant at contact using structured intervention guide (Additional file 2) comprising educational components to clarify discrepancies in medication use following the response to MMAPS questions, the non-pharmacological lifestyle recommendations, as well as encouraging the use of reminders among others. Details of prescribed regimen and clinical outcome parameters at contact and the subsequent 2-months clinic appointment were retrieved from patient’s medical record using data collection form.

### Pretest and validation

The instruments were assessed for content validity by two physicians and two pharmacists chosen from the study sites. Ten randomly selected newly diagnosed hypertensive patients chosen from the Specialist Hospital, Sokoto were used for face validity assessment to ascertain the appropriateness of sampling and recruitment procedure. Feedback from the pretest and validity assessment led to some modifications including rephrasing of closed-ended questions as open-ended questions or ranked variables to ensure comprehension and clarity of intention by the participants.

### Data analysis

Data obtained were analysed using Statistical Package for Social Science software version 22.0. Data were summarised using frequency, percentage, 50th percentile and mean ± standard deviation. Chi-square test was used to evaluate association between socio-demographic variables and adherence to antihypertensive medication and lifestyle recommendations. Student’s t-test was used to investigate relationship between treatment adherence and blood pressure at *p* < 0.05.

## Results

**Fig. 1:**
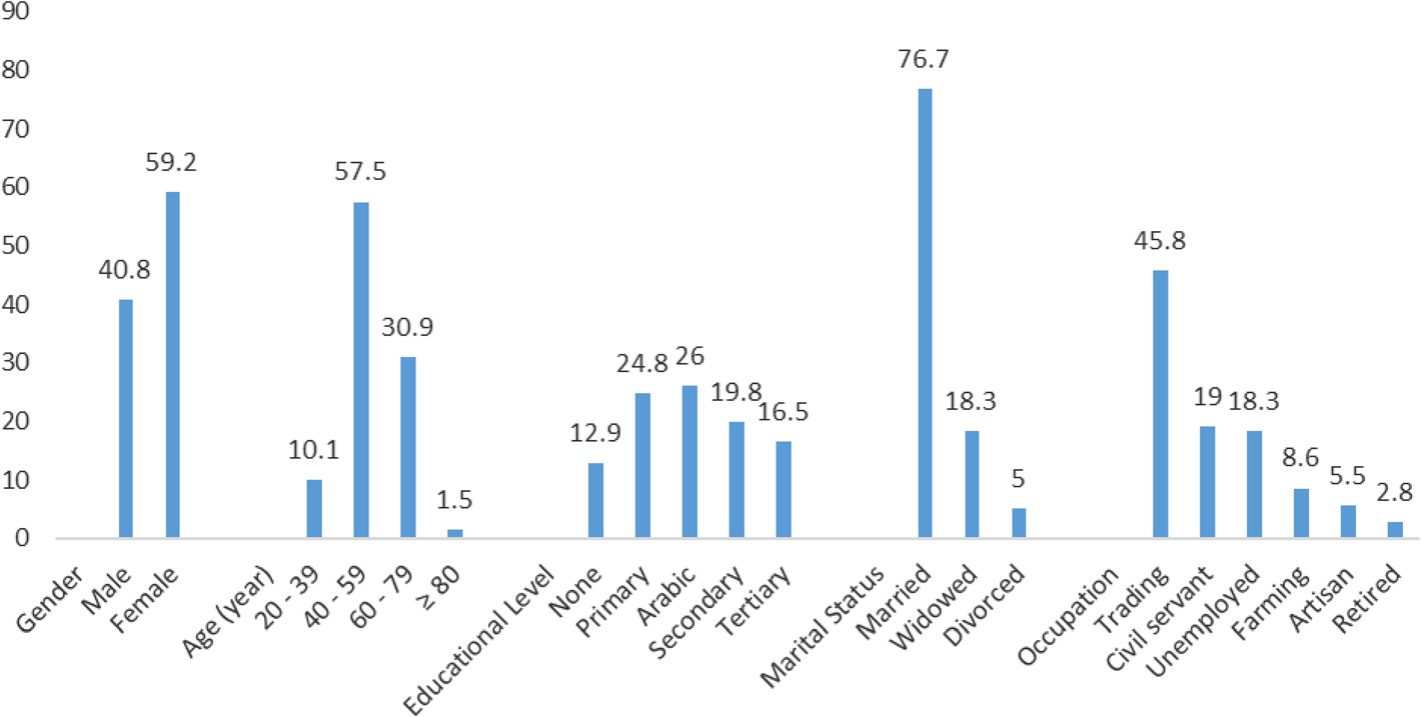
Socio-demographic characteristics of participants (Socio-demographic variables versus percent)

Out of the 624 patients approached for participation within the study period, 605 consented and completed the study, giving a response rate of 97%. There were more females 358 (59.2%) compared to males, 247 (40.8%). The mean age was 54.5 ± 11.4 years, with majority 348 (57.5%) in the age range of 40–59 years (Fig. 1). Forty-three (7.1%) were diagnosed for < 1 year; 430 (71.1%) for 1–5 years; 122 (20.2%) for 6–10 years and 10 (1.7%) patients have been diagnosed for 11–15 years. Summarily, 473 (78.2%) had ≤5 years duration of hypertension and 132 (21.8%) with > 5 years duration.

Response to modified Morisky Adherence Predictor Scale showed that 54 (8.9%) participants had total scores < 1 indicating optimal adherence to antihypertensive medications, while 551 (91.1%) had scores ≥1 suggesting non-adherence (Table 1). Reasons for non-adherence to prescribed medications were mentioned in different combinations to include forgetfulness (404; 35.2%), dose omission (370; 32.2%), side effects (157; 13.7%), non-affordability of medication costs (95; 8.3%), dislike for medication (74; 6.4%), intentional decision to take medicine when desired (34; 2.9%), preference of herbal medicine to conventional drugs (8; 0.7%), while seven (0.6%) mentioned too many drugs to take as reason. Patients with at least a secondary school education had significantly better medication adherence than those with elementary/primary education. Also, adherence is significantly higher among patients on at least 2-medicines compared to patients on monotherapy (Table 2). Patients with duration of hypertension ≤5 years had better medication adherence (66.7%) compared to those (33.3%) with > 5 years (Chi-square (X^2^) = 4.61, *p* = 0.032). Evaluation of adherence to 4-domains of non-pharmacological lifestyle modifications is shown in Table 3. Reasons for non-engagement in exercise were cited in different combinations as lack of time/busy schedule (94; 50.8%), tiredness always (48; 25.9%), dislike for exercise (38; 20.5%), and 5 (2.7%) mentioned fear of being seeing by others. The remainder gave no reason. Adherence to exercise among patients of different educational status was in the order of Arabic education (37; 25.7%) > tertiary education (36; 25.0%) > secondary (30; 20.8%) > primary education (28; 19.4%) > no formal education (13; 9.0%), X^2^ = 12.67, *p* = 0.013. Significant association also exists between educational status and adherence to cigarette smoking cessation (X^2^ = 14.68, *p* = 0.005).

**Table 1:**
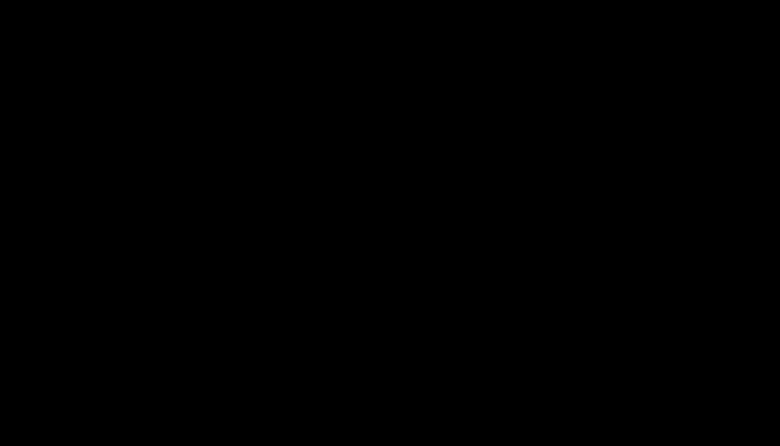
Participants’ response to the 9-item modified Morisky Adherence Predictor Scale (*n* = 605). This table been removed because the authors have not obtained a licence to use the Morisky Medication Adherence Scale-8 (MMAS-8). The results presented in this table are available by contacting the authors

A “Yes” response is assigned a score of “1” suggesting non-adherence and “No”/Never, a score of “zero” indicating better adherence behaviour. Complete responses to all the 9-item questions were considered for distribution into the binary categories of adherence versus non-adherence, *n* number

**Table 2:** Association between relevant categorical variables and medication adherence assessed using modified Morisky Adherence Predictor Scale

Socio-demographic characteristics	Adherence (Score < 1)	Non-adherence (Score ≥ 1)	X^2^	*P*-value
Sex	N (%)	N (%)		
Male	26 (48.1)	221 (40.1)	1.316	0.251
Female	28 (51.9)	330 (59.9)		
Age in years				
20–39	5 (9.3)	56 (10.2)	0.959	0.811
40–59	32 (59.3)	316 (57.4)		
60–79	17 (31.5)	170 (30.9)		
≥ 80	0 (0.0)	9 (1.6)		
Educational Level				
None	2 (3.7)	76 (13.8)	12.759	0.013*
Arabic education	11 (20.4)	146 (26.5)		
Primary education	10 (18.5)	140 (25.4)		
Secondary education	17 (31.5)	103 (18.7)		
Tertiary education	14 (25.9)	86 (15.6)		
Occupation				
Unemployed	14 (25.9)	97 (17.6)	6.579	0.254
Business/Trading	21 (38.9)	256 (46.5)		
Civil servants	13 (24.1)	102 (18.5)		
Retired	2 (3.7)	15 (2.7)		
Farming	1 (1.9)	51 (9.3)		
Artisan	3 (5.6)	30 (5.4)		
Number of antihypertensive medication				
1	14 (25.9)	66 (12.0)	10.381	0.006*
2	22 (40.7)	206 (37.4)		
≥ 3	18 (33.3)	279 (50.6)		

X^2^ = Chi Square, Level of significance *p* < 0.05, *Significant difference with Chi-square

**Table 3:** Response to the 4-domains of non-pharmacological lifestyle recommendations

Domains of non-pharmacological lifestyle recommendations	Yes, n (%)	No, n (%)
Engage in cigarette smoking	74 (12.2)	531 (87.8)
*If yes*, Sometimes	69 (93.2)	
All the times	5 (6.8)	
Engage in alcohol intake	125 (20.7)	480 (78.3)
*If yes*, Sometimes	122 (97.6)	
All the times	3 (2.4)	
Engage in exercise at least three times a week	144 (23.8)	461 (76.2)
*If yes*, brisk walking at least 30 min a day	113 (78.5)	
Hawking goods	13 (9.0)	
Jogging	11 (7.6)	
Skipping rope	4 (2.8)	
Farming	3 (2.1)	
Engage in salt intake	444 (73.4)	161 (26.6)
*If yes*, Rarely	69 (15.5)	
Sometimes	341 (76.8)	
Often	22 (5.0)	
Always	12 (2.7)	
Overall score distribution for lifestyle adjustments	Number (%)	
0	36 (6.0)	
1	170 (28.1)	
2	294 (48.6)	
3	74 (12.2)	
4	31 (5.1)	
Cut-off	Number (%)	Remark
Total score < 1	36 (6.0)	Optimal adherence
Total score ≥ 1	569 (94.0)	Non-adherence

Total avoidance i.e. “No” to smoking, alcohol and dietary salt intake, as well as engaging in exercise for at least three days per week is considered optimal adherence with a score of zero. Overall adherence score to lifestyle adjustments is the sum total of scores from all the 4-domains, *n* = number

Participants’ perception of their hypertensive illness showed that 479 (79.2%) had score between 0 and 50% indicating illness perception as “benign”, while 126 (20.8%) had score between 51 and 100% suggesting illness perception as “threatening”. Beliefs about antihypertensive medication among participants showed that 558 (92.2%) had score > 50% indicating “stronger” beliefs about necessity for antihypertensive medication, and 251 (41.5%) with score > 50% suggesting “stronger” concern about side effects of medication (Table 4).

**Table 4:** Participants’ belief about hypertensive medications (*n* = 605)

Statements	SA	A	U	D	SD	50th Percentile
Necessity Domain	n (%)	n (%)	n (%)	n (%)	n (%)	
My health at present depends on my hypertensive medications	219 (36.2)	297 (49.1)	13 (2.1)	26 (4.3)	50 (8.3)	4
My life would be impossible without my hypertensive medications	23 (3.8)	57 (9.4)	86 (14.2)	187 (30.9)	252 (41.7)	2
Without my hypertensive medications, I would be very ill	75 (12.4)	166 (27.4)	87 (14.4)	131 (21.7)	146 (24.1)	3
My health in the future will depend on my hypertensive medications	123 (20.3)	259 (42.8)	74 (12.2)	72 (11.9)	77 (12.7)	4
My hypertensive medications protect me from becoming worse	254 (42.0)	240 (39.7)	28(4.6)	52 (8.6)	31 (5.1)	4
Concern Domain						
Having to take hypertensive medications worries me	167 (27.6)	183 (30.2)	24 (4.0)	88 (14.5)	143 (23.6)	4
I sometimes worry about the long term effects of my hypertension medication	14 (2.3)	63 (10.4)	164 (27.1)	173 (28.6)	191 (31.6)	2
My hypertensive medications disrupts my life	9 (1.5)	38 (6.3)	119 (19.7)	188 (31.1)	251 (41.5)	2
I sometimes worry about becoming too dependent on my hypertensive medications	22 (3.6)	98 (16.2)	202 (33.4)	137 (22.6)	146 (24.1)	3
Cut off			n (%)	Remark		
Necessity Domain						
Score > 50%			558 (92.2)	“Stronger” belief		
Score ≤ 50%			47 (7.8)	“Weaker” belief		
Concern Domain						
Score > 50%			251 (41.5)	“Stronger” concern		
Score ≤ 50%			354 (58.5)	“Weaker” concern		
Differential						
Score > 0			497 (82.1)	“Stronger” belief about necessity		
Score < 0			103 (17.0)	More concern for side effect/adverse effect		
Score = 0			5 (0.8)	Equal concern for necessity and side effect		

Strongly Agree (SA) = 5, Agree (A) = 4, Uncertain (U) = 3, Disagree (D) = 2, Strongly Disagree (SD) = 1, Maximum obtainable score for the necessity domain = 25; Maximum obtainable score (concern domain) = 20; % score = individual score ÷ maximum obtainable score × 100, Differentials = % score in the necessity domain minus % score in the concern domain, *n* = number

Mean systolic blood pressure at contact was 149.6 ± 22.5 mmHg versus 134.2 ± 15.8 mmHg at 2-months with a 10% reduction. Mean diastolic blood pressure at contact was 87.0 ± 12.0 mmHg versus 80.2 ± 10.4 mmHg at 2-months indicating an 8% reduction. Details of relationships between treatment adherence and blood pressure are shown in Table 5. Components of pharmacist-led patient-specific adherence education were summarily described in Table 6. The order of antihypertensive prescribing preference for patients were calcium channel blockers (492; 32.3%) > angiotensin-converting enzyme inhibitors (394; 25.9%) > diuretics (322; 21.2%) > centrally-acting alpha adrenoceptor blockers (172; 11.3%) > angiotensin receptor blockers (78; 5.1%) > beta blockers (45; 2.9%) > alpha-adrenoceptor blockers (18; 1.2%). Participants with comorbidities/complications were 202 (33.4%), while 403 (66.6%) had no comorbidity. The most common comorbidity was diabetes mellitus (143; 70.8%), others include arthritis (30; 14.9%), heart failure (19; 9.4%), peptic ulcer disease (7; 3.5%) and 3 (1.5%) had benign prostate hyperplasia. Adherence to medication and the 4-domains of non-pharmacological lifestyle modifications was generally better among patients without comorbidity compared to those with comorbidities, though not statistically significant (*p* > 0.05). Thirty-six (66.7%) patients without comorbidity versus 18 (33.3%) with comorbidities were adherent to prescribed medication (*p* = 0.993).

**Table 5:** Association between treatment adherence and blood pressure among patients (*n* = 605)

Evaluation parameters		Number	Baseline SBP (mmHg) Mean ± SD	Baseline DBP (mmHg) Mean ± SD	2-month SBP (mmHg) Mean ± SD	2-month DBP (mmHg) Mean ± SD
Medication	Adherence	54	122.9 ± 11.4	78.4 ± 8.4	121.2 ± 11.0	75.1 ± 10.8
	Non-adherence	551	152.2 ± 21.6	87.8 ± 11.6	135.5 ± 15.7	80.7 ± 10.3
			*P* = 0.000*	*P* = 0.000*	*P* = 0.000*	*P* = 0.000*
Cigarette smoking cessation	Adherence	531	148.4 ± 22.3	87.0 ± 11.5	133.6 ± 15.3	80.2 ± 10.3
	Non-adherence	74	158.1 ± 21.7	86.6 ± 12.4	138.3 ± 18.9	80.4 ± 11.4
			*P* = 0.000*	*P* = 0.792	*P* = 0.018*	*P* = 0.877
Alcohol cessation	Adherence	480	149.2 ± 23.2	87.2 ± 11.9	134.1 ± 15.3	80.6 ± 10.4
	Non-adherence	125	150.9 ± 19.3	86.1 ± 12.4	134.4 ± 16.8	78.8 ± 10.5
			*P* = 0.447	*P* = 0.420	*P* = 0.839	*P* = 0.079
Exercise regimen	Adherence	144	145.7 ± 21.3	86.0 ± 10.4	133.4 ± 15.5	79.4 ± 10.2
	Non-adherence	461	150.8 ± 22.7	87.2 ± 12.0	134.4 ± 16.0	80.5 ± 10.5
			*P* = 0.016*	*P* = 0.280	*P* = 0.506	*P* = 0.277
Dietary salt restrictions	Adherence	161	147.4 ± 22.5	86.4 ± 11.0	134.7 ± 15.0	80.2 ± 10.8
	Non-adherence	444	150.4 ± 22.4	87.2 ± 11.9	134.0 ± 16.2	80.3 ± 10. 4
			*P* = 0.636	*P* = 0.497	*P* = 0.624	*P* = 0.909
Overall lifestyle modification	Adherence	36	147.9 ± 21.3	85.2 ± 9.6	134.8 ± 15.5	81.0 ± 10.7
	Non-adherence	569	149.7 ± 22.6	87.1 ± 11.8	134.2 ± 15.9	80.2 ± 10.4
			*P* = 0.145	*P* = 0.357	*P* = 0.817	*P* = 0.637

*SBP* Systolic Blood Pressure, *DBP* Diastolic Blood Pressure, *SD* Standard deviation, *Significance difference with student’s t-test at *p* < 0.05

**Table 6:** Summary of pharmacist-led patient-specific adherence education

Adherence Education Component	Frequency	Percent
Specific medication adherence improvement strategy (*n* = 2004)		
Provision of clear writing instruction for prescribed regimen	605	30.2
Use of buddy/companion reminder	605	30.2
Advice on measure to take when miss medication dose	461	23.0
Proactive approach to side effect management	245	12.2
Encourage purchase of generic alternative(s)/cheaper brand	88	4.4
Lifestyle adjustment counseling (*n* = 1779)		
Encourage proactive engagement in tolerable level of exercise	605	34.0
Encourage increased intake of fruits, vegetables and low dairy products	533	30.0
Encourage proactive engagement in dietary salt intake reduction	446	25.1
Guidance and counselling on alcohol intake cessation	122	6.9
Provision of guidance and counseling on cigarette smoking cessation	73	4.1
Educational intervention on self-management measure(s) (*n* = 1169)		
Reinforcement of the benefits of blood pressure record keeping as self-care measure	605	51.8
Reinforcement of the inherent importance of routine blood pressure monitoring	564	48.2

Multiple responses were observed in many instances

## Discussion

In this study, less than one-tenth (8.9%) of the patients were optimally adherent to antihypertensive medications. The relatively low medication adherence rate may partly be linked to strict adherence cut-off compared to adherence definitions in previous studies [28, 29, 38, 39]. However, the low level of health literacy among the people in northern part of the country compared to other region may also be a contributing factor for the dismally low medication adherence rate [40, 41]. The two tertiary hospitals used in this study were located in Sokoto state, which is one of the northern states in Nigeria with the highest population of people with low adult health literacy [40–42]. Studies have shown that low health literacy among patients may lead to poor comprehension of treatment regimen, as well as low level of awareness on the need to adopt and maintain healthy lifestyle recommendations, thereby predisposing to poorer health outcome [40, 41, 43].

Forgetfulness top the list of reasons for medication non-adherence, and this is consistent with previous studies that report unintentional reasons as the most common for medication non-adherence [13, 20, 21, 44]. Asymptomatic nature of hypertension coupled with competing psychosocial demands of individuals may perhaps increase the likelihood to forget or omit medication doses [13, 21]. Therefore, healthcare providers should always pay close attention to patient’s psychosocial and medical needs through non-judgmental questioning approach and reflective listening that will enable identification of individual gap(s) preventing medication adherence. Of the patient-specific adherence education provided in different combinations, more than one-third of the patients benefited from counseling relating to the use of buddy or companion reminder as a proactive measure to assist patients in remembering accurate medication dosing regimen and timing. The significantly better blood pressure outcome among patients who were adherent to antihypertensive medication at baseline/contact and the subsequent 2-month interval compared to their non-adherent counterparts is consistent with previous studies, whereby a direct relationship was established between medication adherence and treatment outcome [10, 12, 45]. Resolution of knowledge deficits in individual patient with focus on different aspects of hypertensive management might have contributed to the resultant positive clinical outcome, though, the likelihood of patients obtaining information from other sources may not be completely excluded. However, the follow-up period of 2-months was carefully considered partly to ensure information retention by patients, as well as allowing for a matching with regular clinic appointment, thereby minimizing the possible bias that may arise through patient’s contact with other providers.

In addition, the significantly better medication adherence among patients on at least 2-medicines, mostly as a co-administered combination compared to those on monotherapy may be in contrast to some studies that report an inverse relationship between number of medicines and adherence [10, 23, 45]. More than 90% strongly belief in the necessity of antihypertensive medications to control their high blood pressure, while more than three-quarters perceived hypertensive illness as “benign”. Studies have indicated that health beliefs and behaviour towards a disease or treatment is succinctly influenced by the extent to which individuals believe that they are susceptible to the disease, how severe they believe the disease is, as well as the benefits they stand to gain [21, 22, 46]. It is worthy of note to mention that patients with ≤5 years duration of hypertension and those without complications or comorbidity had better treatment adherence compared to those with > 5 years duration or with comorbidity/complication. Thus, the perceived confidence in prescribed treatment regimen by participants may serve as impetus for healthcare provider to further channel adherence education and counseling towards reinforcing the beliefs and confidence, especially at the early stage of hypertension management, so that the expected outcomes may be largely accomplished.

The overall low rate of adherence to the 4-domains of lifestyle recommendations among participants is a call for concern considering the proven benefits of healthy lifestyle habits in reducing the risk of cardiovascular complications among hypertensive patients [7, 47, 48]. The systolic blood pressure of patients who were adherent to lifestyle recommendations especially cigarette smoking cessation and exercise were significantly better than those who were non-adherent. This therefore underscore the need for redoubled efforts on sensitization and enlightenment of patients to continuously adopt healthy lifestyle habits so as to ensure better blood pressure control. Chobanian et al. (2003) states that lifestyle modifications are fundamentally essential for patients with hypertension and should form an integral part of the disease management [48]. Lack of time top the list of reasons cited by patients for non-engagement in exercise, however, irrespective of the patient’s busy schedule, individuals should always create time to engage in a form of exercise such as brisk walking that will enhance efficient blood circulation [15, 16].

Although, this study provides insight and deeper understanding on the extent of adherence to antihypertensive medication and lifestyle recommendations, as well as beliefs and perception about hypertension and its management. It is however limited by inherent drawbacks with self-report method which include over- or under-report of adherence, as well as patient’s recall bias [49]. Self-report measure using a non-judgmental and non-threatening approach remains a reliable and widely applicable method for assessing treatment adherence in clinical settings [50]. The relatively short follow-up period and absence of a distinct control group also constitute limitations to this study. Thus, future intervention study may need to closely look into these gaps and other relevant factors in order to ensure far-reaching conclusions on the effect of patient-specific adherence education on blood pressure outcome.

## Conclusion

It can be concluded that the overall adherence to antihypertensive medication and the 4-domains of lifestyle modifications is suboptimal. Forgetfulness and busy schedule top the list of reasons for medication and exercise non-adherence, respectively. More than three-quarters have “stronger” belief about the necessity of prescribed antihypertensive regimen in ensuring adequate blood pressure control. Adherent patients have significantly better blood pressure outcome than their non-adherent counterparts. Thus, there is generally a need for continuous patient-specific adherence education and counseling for hypertensive patients in order to ensure better therapeutic outcomes.

## Additional files

Additional file 1: Questionnaire for patients’ interview and retrospective data collection form. (DOC 201 kb)

Additional file 2: Structured intervention guide. (DOC 40 kb)

## References

[1] World Health Organisation-International Society of Hypertension (2003). WHO-ISH statement on management of hypertension. J Hypertens.

[2] World Health Organisation. Global Status Report on Non-communicable Diseases 2010. Geneva: WHO. p. 2011. http://who.int/nmh/publications/ncd_report_full_en.pdf. Accessed 15 July 2017

[3] World Health Organization (2013). A global brief on hypertension: silent killer, global public health crises.

[4] Anastase D, André P, Walinjom FT, Alain M, Charles K, Joseph A, et al. Prevalence, awareness, treatment and control of hypertension in a self-selected sub-Saharan African urban population: a cross-sectional study. BMJ Open. 2012;2:e001217–7. 10.1136/bmjopen-2012-001217 PMC3433777 22923629

[5] Marleen H, Ferdinand W, Marijke R, Lizzy M, Tanimola M, Ingrid H (2011). Hypertension in sub-Saharan Africa: cross-sectional surveys in four rural and urban communities. PLoS One.

[6] World Health Organisation (2005). Facing the facts: the impact of chronic disease in Nigeria.

[7] Iloh GUP, Amadi AN, Okafor GOC, Ikwudinma AO, Odu FU, Godswill-uko EO (2014). Adherence to lifestyle modifications among adult hypertensive Nigerians with essential hypertension in a primary care clinic of a tertiary hospital in resource-poor environment of eastern Nigeria. Br J Med Med Res.

[8] Ogah OS, Rayner BL (2013). Recent advances in hypertension in sub-Saharan Africa. Heart.

[9] Mezue K (2014). The increasing burden of hypertension in Nigeria - can a dietary salt reduction strategy change the trend?. Perspect Public Health.

[10] Thomas B, Philip G, Brian N, Feride FT (2006). Relationship of blood pressure control to adherence with antihypertensive monotherapy in 13 managed care organizations. J Manag Care Pharm.

[11] Akpa MR, Alasia DD, Emem-Chioma PC (2008). An appraisal of hospital based blood pressure control in Port Harcourt. Nigeria Niger Health J.

[12] Luther C (1991). Improving compliance and increasing control of hypertension: needs of special hypertensive populations. Am Heart J.

[13] Karakurt P, Kaşikçi M (2012). Factors’ affecting medication adherence in patients with hypertension. J Vasc Nurs.

[14] Svetkey LP, Erlinger TP, Vollmer WM, Feldstein A, Cooper LS, Appel LJ (2005). Effect of lifestyle modifications on blood pressure by race, sex, hypertension status, and age. J Hum Hypertens.

[15] Xin X, He J, Frontini MG, Ogden LG, Motsamai OI, Whelton PK (2001). Effects of alcohol reduction on blood pressure a meta-analysis of randomized controlled trails. Hypertension.

[16] Appel LJ, Champagne CM, Harsha DW (2003). Effects of comprehensive lifestyle modification on blood pressure control: main results of the PREMIER clinical trial. JAMA.

[17] Wolf-Maier K, Cooper RS, Kramer H, Banegas JR, Giampaoli S, Joffres MR (2004). Hypertension treatment and control in five European countries, Canada, and the United States. Hypertension.

[18] World Health Organization (2003). Adherence to long-term therapies: Evidence for Action.

[19] Al-Ramahi R (2015). Adherence to medications and associated factors: a cross-sectional study among Palestinian hypertensive patients. J Epidemiol Glob Health.

[20] Osterberg L, Blaschke T (2005). Adherence to medication. N Engl J Med.

[21] Meinema JG, van Dijk N, Beune EJ, Jaarsma DA, van Weert HC, Haafkens JA (2015). Determinants of adherence to treatment in hypertensive patients of African descent and the role of culturally appropriate education. PLoS One.

[22] Kang CD, Tsang PP, Li WT, Wang HH, Liu KQ, Griffiths SM (2015). Determinants of medication adherence and blood pressure control among hypertensive patients in Hong Kong: a cross-sectional study. Int J Cardiol.

[23] Osamor PE, Owumi BE (2011). Factors associated with treatment compliance in hypertension in Southwest Nigeria. J Health Popul Nutr.

[24] Kearney PM, Whelton M, Reynolds K, Muntner P, Whelton PK, He J (2005). Global burden of hypertension: analysis of worldwide data. Lancet.

[25] Kettani FZ, Dragomir A, Côté R, Roy L, Bérard A, Blais L (2009). Impact of a better adherence to antihypertensive agents on cerebrovascular disease for primary prevention. Stroke.

[26] Neal B, MacMahon S, Chapman N (2000). Effects of ACE inhibitors, calcium antagonists and other blood pressure lowering drugs: results of prospectively designed overviews of randomized trials. Blood pressure lowering treatment Trialists collaboration. Lancet.

[27] Burnier M (2006). Medication adherence and persistence as the cornerstone of effective antihypertensive therapy. Am J Hypertens.

[28] Fernandez-Arias M, Acuna-Villaorduna A, Miranda JJ, Diez-Canseco F, Malaga G (2014). Adherence to pharmacotherapy and medication-related beliefs in patients with hypertension in Lima, Peru. PLoS One.

[29] Iloh GUP, Ofoedu JN, Njoku PU, Agwu N, Amadi AN, Godswill-Uko EU (2013). Medication adherence and blood pressure control amongst adults with primary hypertension attending a tertiary hospital primary care clinic in eastern Nigeria. Afr J Prim Health Care Fam Med.

[30] Ramli A, Ahmad NS, Paraidathathu T (2012). Medication adherence among hypertensive patients of primary health clinics in Malaysia. Patient Prefer Adherence.

[31] The Creative Research Systems. The complete survey software solution since 1982. Research Aids. https://www.surveysystem.com/sample-size-formula.htm. Accessed 10 Feb. 2017.

[32] Morisky DE, DiMatteo MR (2011). Improving the measurement of self-reported medication nonadherence: final response. J Clin Epidemiol.

[33] Gardner M, Boyce RW, Herrier RN (1994). Pharmacist-patient Consultation Program (PPCP- Unit 3): Counseling to Enhance Compliance. National Healthcare Operations.

[34] Adisa R, Fakeye TO (2014). Treatment non-adherence among patients with poorly controlled type 2 diabetes in ambulatory care settings in southwestern Nigeria. Afr Health Sci.

[35] Adisa R, Fasanmade AO, Fakeye TO (2011). Medication adherence among ambulatory patients with type 2 diabetes in a tertiary health care setting in southwestern Nigeria. Pharm Pract.

[36] Broadbent E, Petrie KJ, Main J, Weinman J (2006). The brief illness perception questionnaire. J Psychosom Res.

[37] Horne R, Weinman J, Hankins M (1999). The beliefs about medicines questionnaire: the development and evaluation of a new method for assessing the cognitive representation of medication. Psychol Health.

[38] Yusuff KB, Alabi A (2007). Assessing patient adherence to antihypertensive drug therapy: can a structured pharmacist conducted interview separate the wheat from the chaff?. Int J Pharm Pract.

[39] DiMatteo M, Giordani P, Lepper H (2002). Patient adherence and medical treatment outcomes: a meta-analysis. Med Care.

[40] Adekoya-Cole TO, Akinmokun OI, Enweluzo GO, Badmus OO, Alabi FO (2015). Poor health literacy in Nigeria: causes, consequences and measures to improve it. Nig. Q. J Hosp Med.

[41] Oladunjoye AO, Adebiyi AO, Cadmus EO, Ige OK, Oladunjoye OO (2013). Health literacy amongst tuberculosis patients in a general hospital in north Central Nigeria. J Community Med Primary Health Care.

[42] Report of the National Literacy Survey. The National Bureau of Statistics and National Commission for Mass Literacy. 2010. http://www.nigerianstat.gov.ng. Accessed 20 Aug. 2017.

[43] Onotai LO (2008). A review of the impact of the health literacy status of patients on health outcomes. The Nigerian Health Journal.

[44] Adisa R, Alutundu MB, Fakeye TO (2009). Factors contributing to nonadherence to oral hypoglycemic medications among ambulatory type 2 diabetes patients in southwestern Nigeria. Pharm Pract.

[45] Krousel-Wood M, Thomas S, Muntner P, Morisky D (2004). Medication adherence: a key factor in achieving blood pressure control and good clinical outcomes in hypertensive patients. Curr Opin Cardiol.

[46] Alhewiti A (2014). Adherence to long-term therapies and beliefs about medications. Int J Family Med.

[47] Teo K, Lear S, Islam S, Mony P, Dehghan M, Li W (2013). Prevalence of a healthy lifestyle among individuals with cardiovascular disease in high-, middle- and low- income countries the prospective urban rural epidemiology (PURE) study. JAMA.

[48] Chobanian AV, Bakris GL, Black HR, Cushman WC, Green LA, Izzo JL (2003). The seventh report of the joint National Committee on prevention, detection, evaluation and treatment of high blood pressure: the JNC VII report. JAMA.

[49] MacLaughlin EJ, Raehl CL, Treadway AK, Sterling TL, Zoller DP, Bond CA (2005). Assessing medication adherence in the elderly; which tools to use in clinical practice. Drugs Aging.

[50] Zeller A, Schroeder K, Peters TJ (2008). An adherence self-report questionnaire facilitated the differentiation between non-adherence and nonresponse to antihypertensive treatment. J Clin Epidemiol.

