# Correction to: Treatment adherence and blood pressure outcome among hypertensive out-patients in two tertiary hospitals in Sokoto, Northwestern Nigeria

**DOI:** 10.1186/s12872-020-01367-7

**Published:** 2020-02-06

**Authors:** Rasaq Adisa, Olumide Ayodeji Ilesanmi, Titilayo Oyelola Fakeye

**Affiliations:** 1 Department of Clinical Pharmacy & Pharmacy Administration, Faculty of Pharmacy, University of Ibadan, Ibadan, Nigeria; 2 Pharmacy Department, Usmanu Danfodiyo University Teaching Hospital, Sokoto, Sokoto state Nigeria

**Correction to: BMC Cardiovasc Disord**

**https://doi.org/10.1186/s12872-018-0934-x**

“After publication of our article [1] the authors became aware that they had not obtained permission to reproduce the questions from the eight-item Morisky’s Medication Adherence Scale.

The table in this Correction replaces Table 1 in our article”.

**Table 1:** Participants’ response to the 9-item modified Adherence Predictor Scale (*n* = 605)

Statements	Yes, n (%)	No, n (%)
Question 1	404 (66.8)	201 (33.2)
Question 2	106 (17.5)	499 (82.5)
Question 3	154 (25.5)	451 (74.5)
Question 4	311 (51.4)	294 (48.6)
Question 5	230 (38.0)	375 (62.0)
Question 6	276 (45.6)	329 (54.4)
Question 7	336 (55.5)	269 (44.5)
Question 8	301 (49.8)	304 (50.2)
Question 9		
Never	192 (31.7)	
Once in a while	248 (41.0)	
Sometimes	161 (26.6)	
Often	4 (0.7)	
Cut–off	Number (%)	Remark
Total score < 1	54 (8.9)	Adherence
Total score > 1	551 (91.1)	Non-adherence

A “Yes” response is assigned a score of “1” suggesting non-adherence and “No”/Never, a score of “zero” indicating better adherence behaviour. Complete responses to all the 9-item questions were considered for distribution into the binary categories of adherence versus non-adherence, *n* number
