# Supplementary material for: Treatment adherence and blood pressure outcome among hypertensive out-patients in two tertiary hospitals in Sokoto, Northwestern Nigeria
Source: BMC Cardiovasc Disord. 2018 Oct 19;18:194. doi: 10.1186/s12872-018-0934-x (PMC6194717; doi:10.1186/s12872-018-0934-x)
Supplement: Supplementary file 1 — Questionnaire for patients’ interview and retrospective data collection form. (DOC 201 kb) [file 12872_2018_934_MOESM1_ESM.doc]

**DEPARTMENT OF CLINICAL PHARMACY AND PHARMACY ADMINISTRATION**

**FACULTY OF PHARMACY**

**UNIVERSITY OF IBADAN**

**QUESTIONNAIRE FOR PATIENTS’ INTERVIEW AND RETROSPECTIVE DATA COLLECTION FORM**

**QUESTIONNAIRE (ENGLISH VERSION)**

***Instruction:***

Dear Respondent,

This questionnaire is strictly for academic research purpose and the response obtained will be treated confidentially. Your sincere cooperation will be required in completing the questionnaire. Thank you for your cooperation.

**SECTION A: SOCIO-DEMOGRAPHIC DATA**

***Instructions:*** *Please tick (****√****) in the box for the most appropriate response*

1a. Age Please specify_____________________________

b Gender Male  Female

c. Marital status Single Married Divorced Widowed Separated

d. Level of education No formal education Primary Secondary Tertiary Arabic education

e. Occupation Unemployed  Business Public servant Retired Others Please specify ________

**SECTION B: ASSESSMENT OF ROUTINE BLOOD PRESSURE MONITORING AND ADHERENCE**

***Instructions:*** *Please tick (****√****) in the box for the most appropriate response and fill in blank where applicable,*

2. When were you told that you have high blood pressure?

Less than one year ago  One year ago Two years ago Three years ago Four years ago five years ago More than five years ago

3a. Routine Blood Pressure monitoring is essential for proper control of High blood pressure, Besides Blood Pressure measurement at the clinic, Have you attempted to have your Blood Pressure checked?

Yes  No

b. If ‘yes’, Where do you have your blood Pressure checked?

At home In the hospital others (please specify)___________

c. If at home Blood Pressure measurement is taken by who?

Yourself  others (please specify)_____________________

4a. Do you keep record of all your home Blood Pressure measurement?

Yes  No

b. If ‘yes’, of what importance is your Blood Pressure measurement record?

Please specify_______________________

5a How often do you have your blood pressure measured?

Daily  once in a Week  twice a week  others (please specify)_______

b. What was your last blood pressure reading? ______________mmHg

6a. Have you been advised by your doctor to go for routine blood pressure check?

Yes  No

b. If ‘yes’, to what extent do you follow this advice?

Never  Sometimes  All the time

c. If ‘Never/Sometimes’, what was the reason for not following this advice?

Please specify_______________________

**SECTION C: ASSESSMENT OF ANTIHYPERTENSIVE MEDICATION USE AND ADHERENCE**

***Instructions:*** *Please tick (****√****) in the box for the most appropriate response and fill in blank where applicable,*

7a. Regular intake of high blood pressure medication is one of the methods of controlling high blood pressure. Were you prescribed any medication to control your high blood pressure? Yes  No

b If ‘yes’, how many high blood pressure medication are you currently taken? ____________________ Medication (s) (please specify)

8. Did you bring along your high blood pressure medication with you today?

Yes  No

8a If yes, can we look at it together, to see how you take each of them (*Show and Tell approach follows e.g. How were you told to take this medication?*

9. What high blood pressure medication are you currently taken?

Mediation Name

Drug A______________________ Drug D______________________

Drug B______________________ Drug E______________________

Drug C______________________ Drug F______________________

10a. In the last one month, did you stop taking any of your blood pressure medications? Yes  No

b. If ‘yes’, what blood pressure medication was stopped?

1______________________ 2______________________

3______________________

c. for what reason(s) was it stopped?

1______________________ 2______________________

3______________________

11. Do you sometimes forget to take your medicine? Yes  No

12. When you travel or leave home, do you sometimes forget to bring along your medicine? Yes  No

13. When you feel like your blood pressure is under control, do you sometimes stop taking your blood pressure medication? Yes  No

14. People sometime miss taking their medication for reason other than forgetting, think over the past three weeks, was there any day when you did not take you blood pressure medication? Yes  No

15 How often do you have difficulty remembering to take all your blood pressure medicine?

Never  once in a while  sometimes  usually  All the time

16a. Have you ever stopped taking your blood pressure medications without telling the doctor? Yes  No

b. If ‘yes’, Why did you stop?

1. I cannot afford the cost  2. Medications are not easily available  3. I do not like to take medications  4. I only take them when I feel that I need them.

5. I do not like the side effects of the medication.  6. I prefer alternative medicine  7. I forget 8. I do not know  9. Advice by spiritual leaders  10. Others (please specify)_____________

17. Does your daily schedule of activities give you time to take your medication? Yes  No

18. Taking medication every day is a real inconvenience for some people. Does taking your blood pressure medicines feels like a burden to you?

Yes  No

19. Do you feel it is important to take you medication, when you don’t experience the symptoms of high blood pressure? Yes  No

20a. Are there other illnesses that you are managing along with hypertension?

Yes  No

b. If ‘yes’, Please specify_______________________

c. What drug are you using (was prescribed) for those illness?

Please specify_______________________

**SECTION D: ASSESSMENT OF PATIENT LIFESTYLE AND ADHERENCE TO LIFESTYLE MODIFICATIONS**

***Instructions:*** *Please tick* ***(√****) in the box for the most appropriate response and fill in blank where applicable,*

21a. Generally, people often engage in some social habit for reason best known to them in spite of report of harmful effects of such habit to their health. Have you ever smoked cigarette? Yes  No

b. If ‘yes’, about how often do you smoke cigarette

Never  Sometimes  All the time

22a. People often use alcohol/alcoholic drinks for recreation purpose. Have you ever drink alcohol? Yes  No

b. If ‘yes’, how often do you drink alcohol

Never  Sometimes  All the time

23a. Have you ever been advised by your doctor about smoking cessation and alcohol intake cessation? Yes  No

b. If ‘yes’, what advice was given? Please Specify ________________

c. To what extent do you follow this advice?

Never  Sometimes  All the time

d. If ‘never’, what was the reason for not following this advice?

Please specify_______________________

24a. Do you participate in any form of physical activity /exercise?

Yes  No

b. If ‘yes’, please specify the type of physical activity (please specify) _____________________________________

If No, state the reason(s) for not engaging in any form of exercise ___________________________________________________________________

25. Salt is an essential ingredient in preparation of various meals. How often do you add salt to food while eating at the table?

Never  Rarely  Sometimes  Often  Always

26a. Do you think that a high salt diet could cause a serious health problem?

Yes No I don’t know

b. If “yes” what sort of problem?

High blood pressure 6 Osteoporosis  Stomach ulcer Don’t know

27a Do you do anything on a regular basis to control your salt or sodium intake?

Yes  No  I don’t know

b. If ‘yes’ what do you do?

1. Avoid /minimize consumption of processed food
2. Look at the salt/or sodium label
3. Do not add salt while eating at the table
4. buy low salt/sodium alternative
5. Use spices other than salt when cooking
6. Others(please specify)_______________________

28

| How often, in the past 30 days, did you eat/drink the following? | Never | Less than 1 time per week | 1-6 times per week | 1-3 times per day | 4 or more times per day |
| --- | --- | --- | --- | --- | --- |
| **Vegetables** (carrots, mushrooms, potatoes, cabbage and spinach, with or without other vegetables etc.) |  |  |  |  |  |
| **Fruit** (apples, bananas, oranges, etc.) |  |  |  |  |  |
| **Legumes and Nuts** (almonds, cashews, walnuts, Beans, refried  beans, baked beans, bean soup, etc.) |  |  |  |  |  |
| **Whole grain** (bread, pasta, rice, cereals etc.) |  |  |  |  |  |
| **Low Fat Dairy** (cheese, milk, yogurt, etc.) |  |  |  |  |  |
| **Sweetened Snack and beverages** ((candy, hamburgers, pizza, fried chicken,  /tacos, cookies, pie, cola, diet cola, energy drinks etc.) |  |  |  |  |  |
| **Red meat and Processed meat** (for example, beef, pork or salt pork, veal, lamb, liver, kidneys, ham, pork chops, ribs, venison, corn beef etc.) |  |  |  |  |  |
| **Food with oils added o**r used in cooking it(moin Shanu, etc) |  |  |  |  |  |
| **Salty Snack and Margarine (regular fat salad dressing** or **mayonnaise**, including on salad and sandwiches, in baked goods, butter etc.) |  |  |  |  |  |

29. Have you ever been advised by your doctor about dietary modification/DASH plan and salt intake reduction? Yes  No

b. If ‘yes’, what advice was given? Please Specify ________________

c. To what extent do you follow this advice?

Never  Once in a while  Sometimes  Usually  All the time

d. If ‘never’, what was the reason for not following this advice?

Please specify_______________________

**SECTION E: ASSESSMENT OF PATIENT’S PERCECPTION ABOUT HYPERTENSION**

30. How well do you feel you understand your high blood pressure?

0 1 2 3 4 5

don’t understand

understand very clearly

31. How much do you experience symptoms from your high blood pressure?

0 1 2 3 4 5

no symptoms many severe symptoms

32. How much does your high blood pressure affect you emotionally? (e.g. does it make you angry, scared, upset or depressed?)

0 1 2 3 4 5

Not at all extremely affected

33. How much do you think your treatment can help control your high blood pressure?

0 1 2 3 4 5

Not at all extremely helpful

34. How long do you think your high blood pressure will continue?

0 1 2 3 4 5 very short time forever

35. How much control do you feel you have over your high blood pressure?

0 1 2 3 4 5

Absolutely extreme amount

no control of control

36. How much your high blood pressure does affect your daily activities?

0 1 2 3 4 5

Not at all severely affects my daily activities

**SECTION F: ASSESSMENT OF PATIENT’S BELIEF ABOUT HYPERTENSION MEDICATION**

Instruction: Beside each of the item statement please mark (x) to indicate you opinion as: SA–Strongly Agree, A –Agree, U –Uncertain, D – Disagree SD – Strongly Disagree

| **S/N** | **BELIEF ABOUT HYPERTENSION MEDICATION** | **SA** | **A** | **U** | **D** | **SD** |
| --- | --- | --- | --- | --- | --- | --- |
| 37 | My health at present depends on my hypertension medicines |  |  |  |  |  |
| 38 | Having to take hypertension medication worries me |  |  |  |  |  |
| 39 | My life would be impossible without my hypertension medication |  |  |  |  |  |
| 40 | Without my hypertension medication I would be very ill |  |  |  |  |  |
| 41 | I sometimes worry about the long term effects of my hypertension medication |  |  |  |  |  |
| 42 | My health in the future will depend on my hypertension medication |  |  |  |  |  |
| 43 | My hypertension medication disrupts my life |  |  |  |  |  |
| 44 | I sometimes worry about becoming too dependent on my hypertension medication |  |  |  |  |  |
| 45 | My hypertension medication protects me from becoming worse |  |  |  |  |  |

**Thank you for answering these questions.**

**DEPARTMENT OF CLINICAL PHARMACY AND PHARMACY ADMINISTRATION**

**FACULTY OF PHARMACY**

**UNIVERSITY OF IBADAN**

**QUESTIONNAIRE (HAUSA VERSION)**

***Ka,idoji:***

*Yakai mai amsa tambayoyi,*

*Wadannan tamboayoyi dasuke acikin wannan takarda anyi sune saboda wannan bincike danakeyi dukkan amsoshin da nasamu zanyi amfani dasu kamar yadda nasamesu. Ina bukatar hadin kanka wurin cike wadanan tambayoyin nagode da hadin kan dakabani.*

**SASHE NA FARKO: YASHAFI MAI CIKE WA**

***Kaidoji****: Ka cika wannan amsar cikin wannan akwatin ta hanyar amfani da alamar maki kamar yada aka tsara.*

1a. Shekara: (Yi bayani).....................

b. Jinsi: Namiji Macce

c. Marar Aure Mai Aure  Wadda Akasaki  Bazawara  Wadanda Sukarabu

d. Matakin Ilimi; Yaki da jahicci Firamari Gaba Da fara mare Jamia

e. Harkaryi: Marar aiki Kasuwanci  Aikin Gwannati  Wanda yabar aiki  Da sauransu(Yi bayani).....................

**SASHE NA BIYU (B): YAKUNSHI CIYON HAWAN JINI DAKUMA YANDA AKE KULA DASHI**

2. Yaushe aka gaya maka kana da hawan jini?

kasa da shekara daya shekara daya shekara biyushekara ukkushekara hudu shekara biyar fiye da shekara biyar

3a. Binciken tsarin hau hawarjini akai–akai yana da muhimmanci wajen shawo kan matsalar hawan jinni. Baya ga haka kataba binciken tsarin hawan jinin ka?

Iyi  A, a

b. In haka ne ina aka taba yima awon hawan jinni?

A. gida A, Asibiti Sauransu (Yi bayani)......................

c. Idan agida ne, wanene yabin ciki tsarin hawan jinin?

Kai da kanka Sauransu (Yi bayani)......................

4a. Shin ko kana da ajiyar-tarihin Gwajin awon jinin da kayi agida?

Iyi  A, a

4b. Idan haka ne minene muhimmancin ajiyar tarihin hawan jinin?

Yi bayani...................................................

5a. Sau nawa kake Gwajin hawan jinin ka?

Kowace Rana  Sau daya a Sati  Saubiyu a Sati Idan kwanayi fiye da haka sai kasa_____________

5b. Minene saka makon awon hawan jinin ka na karshe?.......................mmHg

6a. Likitan ka yabaka shawara karika zuwa awon hawon jini akai akai?

Iyi  A, a

b. Zuwa wane matsayi ka dauki wannan shawara?

Bantabayiba Nakanyi awani lokaci  koyaushe

c. In baka taba ba/ Nakanyi awani lokaci; minene dalilin ka narashin daukar wannan shawara? Sauransu (yi bayani)........................................

**SASHE NA UKKU (C) YAKUNSHI HANYOYIN DA AKE BI DON KIYAYEWA DAYANDA AKE AMFANI DA MAGANIN HAWAN JINI**

***Kaidoji****: Ka cika wannan amsar cikin wannan akwatin ta hanyar amfani da alamar maki kamar yada aka tsara.*

7a. Kiyayewa da kula da cutar hawau jinin itace hanyar da ake iya magance matsalar

Iyi  A, a

7b. Idan haka ne wadannin irin hanyoyi ne ka/ki kabi yanzu? (Yi bayani)......................

Idan akwai wasu hanyoyi sai ka rubuta................................................

8. Ko kana dauke da takardar shedar hawan jinin ka yau?

Iyi  A, a

9. Wace irin hanyace kake bi wajen magance matsalar ka ta hawan jini yanzu?

**Sunan magani**

Magani A..............................magani D....................................

Maganin B............................Magani E....................................

Magani C..............................Magani F....................................

10a. Shin ko watan daya gabata ka taba rashin shan daya daga cikin maganin ka na hawan jini?

Iyi  A, a

10b. Idan ka taba rashin daya daga cikin maganin ka na hawan jinin sai ka rubuta sunan maganin

1........................................ 2...................................3.....................................

10c. Saboda dalilin me kadai na shan maganin

1........................................2...............................3.........................................

11. Shin ko akwai wani lokaci da ka taba man tawa baka sha maganin ba?

Iyi  A, a

12. Lokacin da kayi tafiya ko kabar gida shin ko kataba mantawa da maganin ka da kake sha?

Iyi  A, a

13. Lokacin da kataba jin kasamu saukin ciwon ka na hawan jini koka taba barin shan maganin ka na hawan jini?

Iyi  A, a

14. Wasu lokutta mutane sukan samu kuskure wajen shan maganin su ba don mantuwa ba. Shin ko zaka iya tunawa sati ukku da suka wuce akwai wata rana da baka sha maganin ka na hawan jini ba?

Iyi  A, a

15. Sau nawa ka taba samun matsala wurin tunawa da kasha dukkan maganukkan ka na hawan jini?

Bantababa canba’arasaba Wasu lokutta koda yausheKowane lokaci

16a. Kataba rashin shan maganin ka na hawan jini batare da kagaya wa likita ba?

Iyi  A, a

16b Idan Kataba barin shan maganin miyasa ka daina?

1. Saboda yayimin tsada 2 Saboda ba’a samun maganin ko ina 3. Bana son shan maganin 4 Ina shan su lokacin da naji ina bukatarsu 5 Bani son maganin ya cutardani 6 Nafida son maganin da nake zabi 7 Na kar manta ne 8 Bansani ba 9 Wasu bokaye kebani Shawara Idan akwai sa karubuta___________

17. Shin ko abubuwan da kake yin a yau da kullum sukan baka dama kasha magani?

Iyi  A, a

18. Shan magani kowace rana abune da yake damun mutane kwarai da gaske. Shin ko shan maganin ka na hawan jini yakan dame ka?

Iyi  A, a

19. Shin ko kana ganin yana da kyau mutum yasha maganin koda baiji wani alamu na hawan jini ba?

Iyi  A, a

20. Shin ko akwai wasu cirwatoci da kake kula dasu tare da ciwon kana hawon jini?

Iyi  A, a

b. In akwai sai ka rubuta........................................................................

c. Wane irin magani ne kake amfani dashi...............................................

**SASHE** **NA** **HUDU** (D): **YAKUNSHI TAMBAYOYI AKAN YANDA MUTUN YAKE GUDANAR DA RAYUWARSHI**

***Kaidoji****: Kacika wannan amsar cikin wannan akwatin ta hanyar amfani da alamar maki kamar yada aka tsara*

21a. Duk yawancin mutane sukan kasance da wata dabia wadda sun san tana cutarda lafiyar su ko kataba shan sigari

Iyi  A, a

b. Idan hakane tun yaushe ne ka ke shan sigari?

Bantaba, ba  Wani lokaci Kowane lokaci

22a. Mutane sukan sha giya ko kuma wadan su abubuwan sha da suke dauke da sinadarin giya. shin ko ka taba shan giya?

Iyi  A, a

b. Idan kataba tun yaushe ne kataba shan giyar

Bantaba ba  Wani lokaci  Kowane lokaci

23a. Koka taba samun wata shawara daga likita game da matsalar shan sigari da kuma giya?

Iyi  A, a

b. Idan kataba yi wal\Ce shawarace a kabaka? (Yi bayani).............................................

c. Ta yayane kayi amfani da shawarar da aka baka?

Bantaba ba  Wani lokaci  Kowane lokaci

d. Idan baka taba ba minene dalilin ka na rashin amfani da shawarar da aka baka?

(Yi bayani).................................

24a. Ko kataba shiga cikin wani aiki na motsa jiki? Iyi  A, a

b. Idan ka taba wane irin aikin motsa jiki kake yi? (Yi bayani).................................

25. Gishiri yana daya daga cikin kayan da ake amfani dashi wurin yin abinci. Sau nawa kake kara gishiri akan abincin ka in za kachi?

Bantaba ba Wani lokaci Koda yaushe

26. Shin baka san cewaba saka gishiri mai yawa, kan iya haifarda babbar matsalar ga lafiyar ka?

Iyi  A, a  Bansani ba

b. Idan hakane wace irin matsalace yake kawo wa?

Hawan jini gayanbon ciki Ciwon kasha  Bansani ba

27a. Ko, kanayin wani abu wanda yake taimakamaka wurin kiyaye yadda kake cin gishiri?

Iyi  A, a  Bansani ba

27b. In haka ne yaya kake yi?

1. Ina rage cin abincin da ake sarrafawa
2. Ina kula da gishiri ko kanwar dani ke sha
3. Bana kara gishiri lokacin dani ke cin abinci
4. Nakan yi amfani da abubuwanda ked a gishiri dan kadana cikin su
5. Ni kan yi amfani da tafar nuwa da albasa da sauransu fiye da gishiri wurin yin abinci
6. Idan akwai wani sai kayi bayani........................................................

***Kaidoji***: Kacika wannan amsar cikin wannan akwatin ta hanyar amfani da alamar maki kamar yada aka tsara

| 28. Sau nawane bayan kwana 30 (talatin) kataba ci ko shan daya daga cikin wadannan kayan masu gina jiki? | Bantababa | Fiye da sati daya | Sau daya zuwa sau Shidda a sati | Sau daya zuwa sau ukku a rana | Sau hudu ko feye rana |
| --- | --- | --- | --- | --- | --- |
| **Ganyaye;** Karas, Dankali, Kabeji da suransu tare ko ba tare da wandansu daga cikin wadannan ba. |  |  |  |  |  |
| **Ya, yan Itace;** Aful, banana, abariba aghalima, dabinu, lemu da sauran wasu makamantansu. |  |  |  |  |  |
| **Ire-Iren abinci masu mai dakuma kwaya;**  Kasheu, Wake, Waken soya, Waken Kosai, Waken Miyar Da sauran su |  |  |  |  |  |
| **Ire-Iren abinci wadanda suke dauke da tsaba kamar su;** Shinkafa, Hatsi, Dawa, Masara alikama Maiwa Da sauran su |  |  |  |  |  |
| **Abubuwar masu gina jiki wadan da basuda mai kamar su;** Madara, Yagat, Awara, fura, nono Dawo Da sauran su |  |  |  |  |  |
| **Abubuwar masu gina jiki masu zaki kamar su;** Alewa mai kandi, Kokakola, Fefsi kola Chin-Chin Meat Pie, Da sauran su |  |  |  |  |  |
| **Nama maija da kuma wanda anka sarrafa kamar su;** Naman Shanu, Naman Kilishi, Koda, Zuciya, Awazu, anta, suya, tsinke, tsire Da sauran su |  |  |  |  |  |
| **Abinci da kuma man da ake anfani dashi wurin dafuwar abincin kamar su;** Man Shanu, detu, Man Gyada, Man kwantulora na yaiyan itace. |  |  |  |  |  |
| **Kek din do ake amfani da gishiri wurin yinshi kamar su;** Kek din munar zagayowar haifuwa, Kek din daurin aure, , Salad na gishiri Da sauran su |  |  |  |  |  |

29 Shin ko kataba samun wata shawara daga likitanka game da yadda zaka kula da abubauwan dake ginajiki dakuma yawan gishiri da kakeci

Iyi  A, a

b. Idan kataba; wace irin shawarace akabaka? Sai kayi bayani_________________.

c. Ko tayaya kake gudanar da wannan shawarar da akabaka?

Bantababa Wadansu lukutta Kowane lokaci

d. Idan baka tababa/ Wadansu lukutta; menene dalilinka narashin amfani da shawara?

Sai kayi bayani_____________________.

**SASHE** **NA** **SHIDDA** (**E**)

***Ka,idoji****: Awadannan tamboyin da zasu biyo ana bukatar da kakewaye duk wata amsa dakaga tafi dace wa da yardarka.*

30. Kamar yaya kake ganin kafahin ci yawan ciwon ka na hawon jini?

Zero [0] [1] daya [2] biyu [3] uku [4] hudu [5] biyar

Bangane ba Nagane Uwarai da gaske

31. Tayaya ne kake gano alamonin hawon jini?

Zero [0] [1] daya [2] biyu [3] ukku [4] hudu [5] biyar

Ba wani alamomi Akwai alamomi da yawa

32. Sau nawa ne rashin lafiyar ka ta hawan jini tataba saka (misali) kayi fashi lo kaji tsoro ko ka fita hankalin ka?

Zero [0] [1] Daya [2] biyu [3] Ukku [4] Hudu [5] Biyar

Babu gabadaya Akwai da yawa

33. yaka keganin Kulawar da kake da ciwon ka na hawan jini zaitai makam wurin magance matsalar

Zero [0] [1] Daya [2] biyu [3] Ukku [4] Hudu [5] Biyar

Babu gabadaya Akwai cutar da yawa

34. Kamar tsawon ware lokaci ne kake ganin ciwon na hawan jini zai ci gaba?

Zero [0] [1] Daya [2] biyu [3] Ukku [4] Hudu [5] Biyar

Cikin lokaci kadan har abada

35. Wane irin matakine kake ganin ka dauka domin magance wannan matsalar ta ka ta hawan jini?

Zero [0] [1] Daya [2] biyu [3] Ukku [4] Hudu [5] Biyar

Gaskiya ba yanda zanyi in magance Akwai hanyoyi da matsalar dama dazan iya magance wannan matsalar

36. Kamar sau-nawa wannan cutar ta ka ta hawan jini ta hanaka gudanar da abubuwan ka na yau da kulum?

Zero [0] [1] Daya [2] biyu [3] Ukku [4] Hudu [5] Biyar

Bai taba hanani ba yakan hana ni gudanar da a bubuwana nay au da kulum

**SASHE NA BAKWAI (F)**

***Ka,idoji*** *Tsakanin kowane irin tambaya da ka ansa kayi kokari kanuna irin amincewar ka tahanyar:* ***SA: Nayarda kwarai, A: Akwai, U: kuwan kwanto, D: Banyarda ba, SD: Banyarda ba kwarai.***

| **S/N** | **YARDA DA MAGANIN CUTAR**  **HAWAN JINI** | **SA** | **A** | **U** | **D** | **SD** |
| --- | --- | --- | --- | --- | --- | --- |
| 37 | Samun lafiyata a yanzu yadogara ne akan maganina na hawan jinni |  |  |  |  |  |
| 38 | Shan manganina na hawan jini yana da muna |  |  |  |  |  |
| 39 | Bazan iya rayuwa ba idan ban sha, magani na nahawan jini ba. |  |  |  |  |  |
| 40 | Idan ban sha maganina na hawan jini ba nakan kamu da rashin lafiya |  |  |  |  |  |
| 41 | Wadan su lokutta na kan samu damuwa saboda tsayon lokacin da maganina na hawan jini yakeyi yana yimin aiki ajikina |  |  |  |  |  |
| 42 | Cigaba da rayuwata ya dogara ne akan maganina da nake sha na Hawan jinni |  |  |  |  |  |
| 43 | Magani na na hawan jini yana kawomin cikas a,rayuwa ta |  |  |  |  |  |
| 44 | Wadan su lokutta nakan samu damuwa saboda dogaro da shan maganina na hawan jinni a koda yaushe |  |  |  |  |  |
| 45 | Shan maganina da nakeyi na hawan jini yakan kara kare ni daga karuwar cutar. |  |  |  |  |  |

***Nagode*** ***da kabana amsar wadannan tambayoyi.***

**RETOSPECTIVE** DATA COLLECTION FORM

| **S/n** | **Hospital number** | **Other disease conditions** | **Antihypertensive prescribed and dosage regimen** | **Other medication(s)** | **Documentation of side effect (s)** | **Average of 2 or 3 consecutive blood pressure values at contact** | **Average of consecutive blood pressure values at 2 months subsequent appointment** |
| --- | --- | --- | --- | --- | --- | --- | --- |
|  |  |  |  |  |  |  |  |
