# Supplementary material for: Treatment adherence and blood pressure outcome among hypertensive out-patients in two tertiary hospitals in Sokoto, Northwestern Nigeria
Source: BMC Cardiovasc Disord. 2018 Oct 19;18:194. doi: 10.1186/s12872-018-0934-x (PMC6194717; doi:10.1186/s12872-018-0934-x)
Supplement: Supplementary file 2 — Structured intervention guide. (DOC 40 kb) [file 12872_2018_934_MOESM2_ESM.doc]

**STRUCTURED INTERVENTION** GUIDE

|  |  |  | |
| --- | --- | --- | --- |
|  |  |  |  |
| 1 | SECTION A: Regular BP Monitoring |  |  |
|  | Education on   1. Blood pressure measurement values 2. Instruments 3. Importance of regular blood pressure measurement/Monitoring 4. Record keeping of blood pressure 5. Importance of record keeping |  |  |
|  | SECTION B:Medication use and Adherence to medications(antihypertensives) |  |  |
|  | 1. Education on Hypertension treatment modalities 2. Counselling on the use of medications  - Medication regimen, missed doses  1. Addressing barriers to non- adherence:   Forgetfulness-use of reminder, daily calendar etc.   - 1. Side effect – Side effect, Cost, drug availability   Encourage report  Management of common ones  Associated with hypertension  Cost - Recommendation switch of brand   1. Addressing interference of other drug with antihypertensive issue 2. Change to other brand or removing the offending drug 3. Addressing barriers to non- adherence:    1. Forgetfulness, 4. Other Medication |  |  |
| 3 | SECTION C: Life style modifications |  |  |
|  | 1. Counselling on smoking and alcohol intake cessation 2. Counselling on cessation of cigarette smoking 3. Counselling on cessation of alcohol intake 4. Counselling on exercise/weight reduction 5. Counselling on reduction on salt intake/DASH diet 6. Encourage DASH diet 7. Telling the patient the harmful effect of alcohol and smoking 8. Encourage smoking and alcohol cessation 9. Education on types of exercise   Assessment of patient potential disability  Encourage regular, moderate exercise appropriate to the patient situation   1. Education on reduction on salt intake/DASH diet   Alert the patient to the danger of high salt diet  Teach the patient how to control salt intake  Encourage DASH diet  High intake of fruits and vegetables, legume, whole grain and low fat dairy product  Reduction in intake of Alcoholic beverages, red meat salty snack and salty processed food. |  |  |
| 4 | SECTION D: Social support |  |  |
|  | 1. Enquire if there is hypertensive club or social support group 2. Education on relevance of joining such group 3. Encourage the patient to join any |  |  |
| 5 | SECTION E :Perception about Hypertension |  |  |
|  | 1. Education on hypertension, causes, its symptoms, and treatment |  |  |
| 6 | SECTION F: Belief about Medication(antihypertensive) |  |  |
|  | 1. Counselling on Medications(antihypertensive) 2. Medication name and dosage 3. Proper uses of medication 4. Counselling on dangers of not taking medications |  |  |
